# When Users Log Mood After Assessment: Temporal-Lag, Discriminant Validity, and the Simplicity Paradox of Single-Label Ecological Momentary Mood Tracking in a Saudi Arabic-Language Digital Mental Health Cohort—A Diagnostic-Accuracy Study

**DOI:** 10.3390/diagnostics16213560

**Published:** 2026-10-29

**Authors:** Abdulmajeed A. Alkhamees

**Affiliations:** Department of Psychiatry, College of Medicine, Qassim University, Saudi Arabia; a.alkhamees@qu.edu.sa

**Keywords:** ecological momentary assessment, mood tracking, screening-positive detection accuracy, convergent-validity signal, Arabic PHQ-9, Arabic GAD-7, discriminant validity, digital mental health, Saudi Arabia, STARD

## Abstract

**Background**: Digital mental health platforms increasingly deploy self-directed single-label mood tracking as an ecological momentary assessment (EMA) tool, often framed as a screening indicator for depression or anxiety. Whether such naturalistic mood logs accurately detect screening-positive PHQ-9 and GAD-7 scores has not been tested at scale against validated Arabic-language reference-standard instruments. Objectives: To evaluate, retrospectively, how accurately naturalistic single-label Arabic mood tracking detects screening-positive scores on the validated Arabic PHQ-9 and Arabic GAD-7 administered on the same platform, and to characterise the convergent-validity signal on continuous scores. **Methods**: This was a retrospective linked-records study of a Saudi Arabic-language commercial digital mental health platform. From 25,555 adult mood-logging users (aged 18 years or older), we assembled three intersection cohorts: depression (n = 4236), anxiety (n = 2449), and both-scales (n = 1825). Nine Arabic mood labels were categorised a priori as negative, positive, or neutral. Operating characteristics with bootstrap 95% confidence intervals were computed for four primary indices; thirteen comparator specifications and four machine-learning models were compared against the best-performing index. AUC differences were evaluated with a DeLong-equivalent bootstrap test. Reporting followed STARD. **Results**: The best-performing index (proportion of negative logs) yielded an AUC of 0.624 (95% CI 0.604–0.642) for screening-positive detection of depression and 0.652 (0.629–0.676) for anxiety, both below the conventional 0.80 benchmark for a clinically useful screening test. Youden-optimal sensitivity and specificity were 0.522 (0.506–0.538) and 0.712 (0.679–0.741) for depression and 0.569 (0.547–0.589) and 0.706 (0.670–0.743) for anxiety. Positive likelihood ratios of 1.81 and 1.93 fell below the 2–5 range that is conventionally associated with even small clinically meaningful shifts in post-test probability. Spearman correlations with continuous scores were modest but stable across bootstrap resamples (ρ = 0.232 for depression, 0.268 for anxiety; both *p* < 0.001). Three structural findings emerged. First, a temporal-sequencing pattern was observed: median lag was +19 days for depression and +25 days for anxiety, with 80.9% and 79.4% of mood logs recorded on or after scale completion, respectively; among users with mood logs recorded before scale completion, AUC was 0.586 (0.554–0.618) for depression and 0.605 (0.569–0.641) for anxiety. Second, genuine discriminant validity was found for the depression-specific composite (Steiger’s Z = 2.82, *p* = 0.005) but not for the anxiety-specific single label (Z = 0.32, *p* = 0.75), reflecting a 2.24% anxiety-label prevalence on this platform. Third, we observed a simplicity-paradox pattern in which added composite complexity either failed to raise cross-validated AUC or destroyed discriminant validity. **Conclusions**: On this platform, single-label Arabic mood tracking shows a modest but statistically reliable retrospective association with screening-positive status on the validated Arabic PHQ-9 and Arabic GAD-7 that does not reach the conventional 0.80 AUC benchmark for a clinically useful screening test; it should not substitute for these validated instruments. It is best treated as a longitudinal engagement and self-monitoring indicator rather than a stand-alone screening tool.

## 1. Introduction

### 1.1. The Diagnostic-Accuracy Assumption in Digital Mood Tracking

Digital mental health platforms have proliferated in the past decade, and among their most widely deployed features is self-directed daily mood tracking [1,2]. Users self-select a mood label—typically from a small discrete set—and the resulting time-stamped log constitutes a naturalistic ecological momentary assessment (EMA) record [3]. Platform-design guidelines and consumer-facing marketing routinely frame naturalistic mood logs as an early-warning or screening indicator for clinically significant depression or anxiety [1,2,4]. That diagnostic-accuracy assumption has substantive implications: if valid, mood tracking could operate as a low-friction screening step that lowers the threshold to help-seeking; if not, it may be misappropriated as a substitute for validated instruments in settings where the latter would be clinically indicated.

Empirically, the assumption has been repeatedly stated but rarely tested at scale against a well-defined reference-standard instrument [5,6]. Studies documenting cross-sectional associations between mood-tracking engagement and app-level engagement or satisfaction [7,8] do not test diagnostic accuracy in the technical sense; they leave the sensitivity, specificity, and likelihood-ratio properties of mood tracking as a screening approach essentially uncharacterised. Direct diagnostic-accuracy studies against PHQ-9/GAD-7 references are especially rare in non-English-language contexts, and to our knowledge have not been reported for the Arabic-speaking Gulf using validated Arabic versions of these instruments.

### 1.2. The Arabic and Platform-Specific Context

Mental disorders are common in Saudi Arabia: the Saudi National Mental Health Survey estimated a lifetime prevalence of any DSM-IV disorder of 34.2% and a 12-month prevalence of 20.2% [9,10], and the 2022 national screening initiative documented persistently low treatment rates alongside substantial community-level distress [11]. Digital mental health platforms operating in Arabic have grown accordingly. The specific Saudi platform studied here (Talbinah) administers the validated Arabic PHQ-9 [12] and Arabic GAD-7 [13] alongside a self-directed nine-label naturalistic mood-tracking feature. Whether the naturalistic mood logs produced by the platform’s users carry meaningful diagnostic-accuracy signal against those validated screening scales in this specific commercial-platform setting is the question this study addresses.

### 1.3. Objectives

Using linked, time-stamped, individual-level records from a single Saudi Arabic-language digital mental health platform, we set out to evaluate the retrospective (concurrent) diagnostic accuracy and convergent validity of naturalistic single-label mood tracking against validated Arabic PHQ-9 (depression) and Arabic GAD-7 (anxiety) scale scores administered on the same platform. We pre-specified an exhaustive sensitivity sweep across alternative index specifications and supervised-learning models to characterise the plausible range of achievable diagnostic accuracy for this data structure on this platform, not only its baseline value. Beyond the headline validity question, we examined three structural properties of naturalistic single-label EMA that have received limited attention in the diagnostic-accuracy literature: the temporal ordering of mood logs relative to formal reference-standard completion; the separability of the depression signal from the anxiety signal (discriminant validity); and the trade-off between overall accuracy and discriminant validity as index complexity is increased.

## 2. Materials and Methods

### 2.1. Design and Setting

**Figure 1:**
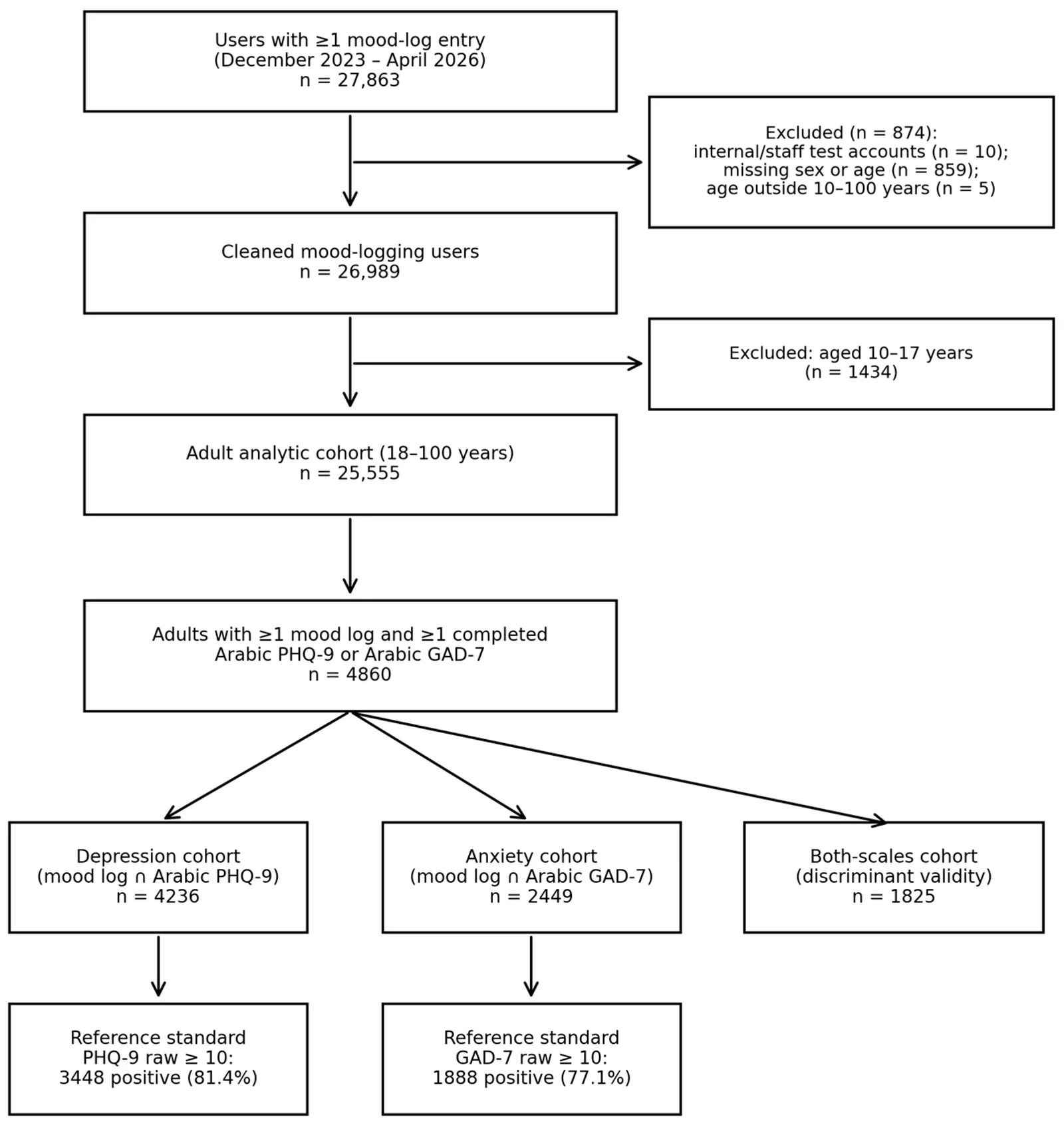
STARD-compliant flow of participants through the study. From 27,863 mood-logging registrants during the extraction window (December 2023–April 2026), 874 were excluded (internal/staff test accounts; implausible age; missing sex/age), and a further 1434 users aged 10–17 years were excluded, yielding a 25,555-user adult analytic cohort. Three intersection cohorts were constructed from users with both mood logs and at least one completed formal-scale administration: depression (n = 4236; positivity 81.4%), anxiety (n = 2449; positivity 77.1%), and both-scales (n = 1825).

This was a retrospective linked-records study using de-identified data from Talbinah, a Saudi Arabia-based Arabic-language commercial digital mental health platform. Mood-log records and formal scale administrations spanned December 2023 to April 2026. Reporting followed the STARD statement for diagnostic-accuracy studies [14] and the STROBE statement for observational epidemiology [15]. The STARD participant-flow diagram is provided as Figure 1.

### 2.2. Data Sources and Platform Workflow (A Priori Disclosure)

Three linked platform tables were used, joined by an SHA-256 pseudonymous user identifier: (i) a user-level demographic table; (ii) a daily mood-log table with one row per (user, timestamp, mood label); and (iii) a psychometric assessment table with one row per (user, scale, attempt, timestamp) and per-attempt raw score, score percentage, and platform-assigned severity band.

The platform workflow is symmetric with respect to the two components under study. Mood logs are self-initiated by the user at any time and do not require a preceding or following scale completion; scale administrations are also self-initiated and do not require a preceding or following mood log. There is no forced funnel that requires either behaviour before the other. This a priori disclosure matters because a forced ordering would confound the temporal analysis in Section 3.4.

### 2.3. Cohort Assembly and Inclusion Criteria

Of 27,863 mood-logging registrants, 26,989 remained after standard exclusions (internal or staff test accounts, implausible age outside 10–100 years, missing sex or missing age). All users aged 10–17 years (n = 1434) were then excluded so that the analysed cohort comprises adults only, giving an adult analytic cohort of 25,555 users. Intersection-cohort inclusion required at least one completed platform administration of the relevant validated Arabic scale AND at least one mood-log entry within the platform’s data window. Users with only one mood log were retained (this is a diagnostic-accuracy study, not a longitudinal engagement study). The three intersection cohorts were: depression (n = 4236), anxiety (n = 2449), and both-scales (n = 1825). Reference-standard positivity at the raw-score ≥ 10 threshold was 81.4% (depression) and 77.1% (anxiety); the Discussion Limitations section addresses the spectrum-effect implications of this high positivity.

### 2.4. Reference-Standard Definition

For each user in each intersection cohort, the reference-standard score was taken from the first-attempt completion of the relevant validated Arabic scale (choice defended in Section 2.10 sensitivity analysis). The primary binary reference-standard threshold was raw score ≥ 10 on both scales. The raw-score threshold ≥ 10 was chosen as the primary reference standard because it is the standard clinical cutoff for at least moderate depression on the PHQ-9 (Kroenke, Spitzer & Williams 2001 [16]) and moderate anxiety on the GAD-7 (Spitzer, Kroenke, Williams & Löwe 2006 [17]), and is directly interpretable across studies. The platform’s own severity-band classification is a downstream categorisation derived from the same raw score; using the raw-score threshold preserves comparability with the international diagnostic-accuracy literature. A positive screen at this threshold is not a clinical diagnosis and requires clinician confirmation to be actionable; the terms “screening-positive” or “positive at the screening threshold” are used consistently throughout. The platform additionally administers a Suicidal Ideation Scale; this instrument falls outside the analytic scope covered by ethics approval T-D-1911 and was excluded from all analyses reported here.

The platform administers the Arabic PHQ-9 in the version psychometrically validated by AlHadi et al. (2017) [12] in a Saudi Arabic-speaking primary-care and community sample (n = 1485), where it demonstrated content equivalence with the English PHQ-9, high internal-consistency reliability (Cronbach’s alpha = 0.857), and criterion-related validity against the Mini International Neuropsychiatric Interview. The platform administers the Arabic GAD-7 in the version psychometrically validated by Sawaya et al. (2016) [13] in a Lebanese Arabic-speaking primary-care sample (n = 128), where it demonstrated content equivalence with the English GAD-7, high internal-consistency reliability (Cronbach’s alpha = 0.874), and criterion-related validity against the Structured Clinical Interview for DSM-IV. We acknowledge that the GAD-7 validation population is Lebanese Arabic-speaking rather than Saudi Arabic-speaking, and that no Saudi-specific psychometric validation of the Arabic GAD-7 exists in the peer-reviewed literature to our knowledge; the cross-national portability of the Arabic GAD-7 psychometrics from Lebanon to Saudi Arabia is therefore an inference rather than an established property, and this is noted as a limitation (Section 4.4). Item-level responses were not accessible in the platform data feed (only aggregate raw score and score percentage per attempt); this constrains what item-level analyses of screening-positive detection accuracy were possible (see Section 2.9 Set D).

### 2.5. Mood Label Classification (A Priori)

Nine distinct Arabic mood labels were present in the platform data. These were categorised a priori by the study author on standard affective-valence conventions: Negative: حزين (Sad), محبط (Frustrated), متعب (Tired), قلق (Anxious), غاضب (Angry)—19.46% of all logs. Positive: سعيد (Happy), راضي (Satisfied)—66.64% of all logs. Neutral: محايد (Neutral), متأقلم (Coping)—13.90% of all logs.

### 2.6. Mood Indices Tested

Four primary candidate indices were pre-specified as the confirmatory analyses: (1) Proportion of negative logs (neg_mood_ratio): user-level count of negative-valence logs divided by total logs. (2) Dominant-mood-negative (binary): the most frequent label is a negative-valence one (ties resolved toward negative). (3) Mood-label entropy (Shannon entropy across the nine labels): information-theoretic diversity measure. (4) Negative-day count ≥3 (binary): user has at least three distinct calendar days with any negative log. Two additional composites were pre-specified for the discriminant-validity sub-analysis: (5) Depression-specific composite (Sad + Frustrated + Tired ratio); (6) Anxiety-specific composite (Anxious ratio initially; extended to Anxious + Frustrated after preliminary inspection revealed Anxious-label prevalence of only 2.24%).

### 2.7. Diagnostic-Accuracy Analyses

For each candidate mood index and each reference-standard construct (depression, anxiety), we computed: area under the receiver-operating characteristic curve (AUC) with 1000-iteration bootstrap 95% confidence intervals; sensitivity, specificity, positive predictive value (PPV), negative predictive value (NPV) at the Youden-optimal threshold, each with bootstrap 95% CI; positive and negative likelihood ratios with bootstrap 95% CI; and Spearman rank correlation with continuous score percentage with bootstrap 95% CI. Between-index AUC comparisons used a DeLong-equivalent bootstrap test (resampling the paired predictions on the same subjects to construct the 95% CI of the AUC difference and the two-sided *p*-value).

Youden-optimal threshold selection was performed in-sample for the reporting table and is transparently flagged as an optimistic operating-characteristic presentation relative to a fully held-out threshold-selection scheme; the 5-fold cross-validated AUC reported in Section 3.6 is the primary held-out generalisation estimate. AUC ≥ 0.80 is a commonly cited benchmark for a clinically useful screening test (Pepe 2003 [18]; Mandrekar 2010 [19]); likelihood-ratio interpretation followed the Jaeschke et al. (1994) [20] conventions (LR+ ≥ 10 large, 5–10 moderate, 2–5 small but sometimes important).

Discriminant validity was tested in the both-scales cohort (N = 1825) using Steiger’s Z-test for the difference between three dependent correlations (Steiger 1980 [21]), specifically: (i) ρ (depression-specific composite, depression score); (ii) ρ (depression-specific composite, anxiety score); and (iii) ρ (depression score, anxiety score). All three correlations are estimated on the same subsample, satisfying the dependent-correlation assumption of Steiger’s Z. A significant Z at α = 0.05 supports discriminant separability. The multivariate-normality assumption is approximate for rank correlations; the observed Z should be read as evidence of separability at the level of dependent correlations rather than a distributional-parametric guarantee.

E-values for the significant regression coefficients (Section 2.8) were computed using the VanderWeele–Ding formulation [22] for both the point estimate and the confidence-interval bound closer to the null. E-values quantify the minimum joint association strength that a hypothetical unmeasured confounder would need with both exposure and outcome to fully explain the observed association away, under the assumption of no measurement error and no further residual confounding; they do not prove absence of residual confounding.

### 2.8. Regression Models

For each construct, an ordinary least-squares regression of continuous score percentage on the primary mood index, adjusted for age (per 10-year increment), sex (male vs. female), and device type (iOS vs. other), was fitted; standardised beta coefficients, VIF, and adjusted R^2^ were reported. Device type was missing for 2.9% of users in the depression cohort, and 4.7% in the anxiety cohort; the primary models used complete cases, and a missing-indicator specification is reported in Section 3.7. The adjustment set (age, sex, and device type) is the demographic-and-access set reliably available on the platform: age and sex are theoretically associated with both mood-logging patterns and reference-standard scores in this population; device type is a crude socioeconomic proxy in a Gulf context where iOS-vs.-Android use correlates with income and urban-rural residence. This adjustment set does not control for symptom severity trajectory, prior treatment, psychosocial stressors, or platform-onboarding funnel effects—the E-values are presented as quantifying, not eliminating, this residual-confounding concern. A parallel logistic regression on binary reference-standard positivity yielded odds ratios with 95% CIs and out-of-fold discrimination (AUC).

### 2.9. Pre-Specified Robustness Sweep—Ceiling Characterisation Within the Pre-Specified Feature Space

To characterise the plausible range of achievable diagnostic accuracy on this data structure, we pre-specified alternative index specifications (Sets A and B) and four machine-learning models (Set C); together with the three other primary indices and the anxiety-specific single label, thirteen comparator index specifications were compared against the primary index. This robustness sweep was based on a written internal analysis protocol drafted before the analyses were executed; it was not registered in a public timestamped registry, and we accordingly use “pre-specified” throughout to accurately reflect the evidentiary status.

Set A (temporal/volatility): mood volatility (SD of numerically coded valence), consecutive-negative-day maximum streak, recent 30-day and 7-day negative-day counts anchored to the scale-completion date, negative-ratio trend slope, positive-to-negative transition count.

Set B (weighted composites): depression-specific composite (Sad + Frustrated + Tired), anxiety-specific composite (Anxious + Frustrated), and a weighted-negative composite (Sad × 3 + Frustrated × 2 + Tired × 1 + Anxious × 1.5 + Angry × 1).

Set C (machine-learning models): Random Forest, LightGBM, XGBoost, and a logistic regression with pairwise interactions, each using the primary index, entropy, log count, the Set A temporal features, and the two binary primary indices as inputs. All models used 5-fold StratifiedKFold cross-validation with out-of-fold AUC reported. Preprocessing (feature scaling) was fitted within each training fold only and applied to the corresponding validation fold; hyperparameter tuning was performed via nested cross-validation on training folds only; feature selection was pre-specified rather than data-driven.

Set D (item-level PHQ-9/GAD-7 mapping) was pre-specified but proved not possible because the platform data feed includes only aggregate raw_score and score_pct per attempt, not individual-item responses.

### 2.10. Sensitivity Analyses

(i) Complete-case analysis versus a missing-indicator specification for the single missing covariate (device type; 2.9% missing in the depression and 4.7% in the anxiety cohort). (ii) Reference-standard-attempt sensitivity: first, last, and all-attempt specifications. (iii) Temporal-window sensitivity: mood-log features computed on the full user timeline versus logs recorded strictly before the reference-standard completion date (any time before, and within 30 and 7 days before). Pre-completion AUCs were estimated in users with at least one pre-completion log; as a secondary specification, all cohort members were retained, and users without pre-completion logs were assigned a negative-log proportion of zero. (iv) Threshold sensitivity: raw ≥ 10 versus platform severity ≥ 40%.

Analyses used Python 3.10.12 with pandas 2.3.3, NumPy 2.2.6, SciPy 1.15.3, statsmodels 0.15.0, scikit-learn 1.7.2, XGBoost 3.2.0 and LightGBM 4.7.0; bootstrap resampling used 1000 iterations with a fixed random seed.

### 2.11. Ethics—Jurisdiction and Regulatory Framework

The study was approved by the Talbinah Research Ethics Committee (reference T-D-1911; approval date 18 March 2026), the standing institutional review body of the Talbinah platform. The Talbinah REC is composed of external clinical and research members and is functionally independent of the platform’s product and commercial decision-making. Qassim University’s Institutional Review Board was formally consulted and issued a written confirmation that the Talbinah REC is the appropriate reviewing body for research using platform-held de-identified records; this confirmation is available on request.

### 2.12. Ethics—Consent Framework

User consent at registration to the secondary use of de-identified data for service improvement and research as part of the Talbinah platform’s Terms of Service, and this registration-based consent covers the research use of de-identified data in the present analysis; no additional individual consent was sought. We acknowledge that platform Terms of Service consent for secondary research use is a lower-friction consent model than opt-in specific consent for each study; this consent framework aligns with CIOMS 2016 International Ethical Guidelines for Biomedical Research (Guideline 12) and with Saudi National Committee of Bio & Medical Ethics guidance on secondary research use of de-identified electronic health records. All data used here were fully de-identified by the platform’s data-governance function before the author accessed them, and no re-identifiable information was accessed at any point in the analysis. A stricter opt-in consent model would be preferable for identifiable data; we note this as a Limitations item.

### 2.13. Ethics—Author’s Relationship with the Platform

The author accessed the de-identified dataset under a formal data-governance agreement with the Talbinah platform. The author is a founder of Talbinah, has a financial interest in the company, and serves as a consultant to it. The Talbinah Research Ethics Committee, which approved this study, is separate from the author: he is not a member of the committee and had no role in its review or decision. The study was performed in accordance with the Declaration of Helsinki.

## 3. Results

### 3.1. Intersection Cohorts and Reference-Standard Characteristics

Depression-intersection: N = 4236; anxiety-intersection: N = 2449; both-scales: N = 1825 (Figure 1). Reference-standard positivity at the raw-score ≥ 10 threshold was 81.4% for depression and 77.1% for anxiety, reflecting a spectrum-effect signal that platform users who self-initiate a formal-scale completion are enriched for symptomatic individuals relative to the general population; operating characteristics derived on this enriched sample will not generalise directly to a general-population screening setting (Discussion Limitations). Mood-log volume per user in the depression cohort was heavily right-skewed: median 3 logs (IQR 1–6, maximum 573); anxiety cohort median 3 (IQR 1–9, maximum 573). Mean age was 27.6 years (depression) and 27.2 years (anxiety); 77.7% and 78.8% were female. Cohort-to-analytic-cohort demographic comparisons are provided in Supplementary Table S1.

### 3.2. Primary Diagnostic Accuracy (Table 1)

**Figure 2:**
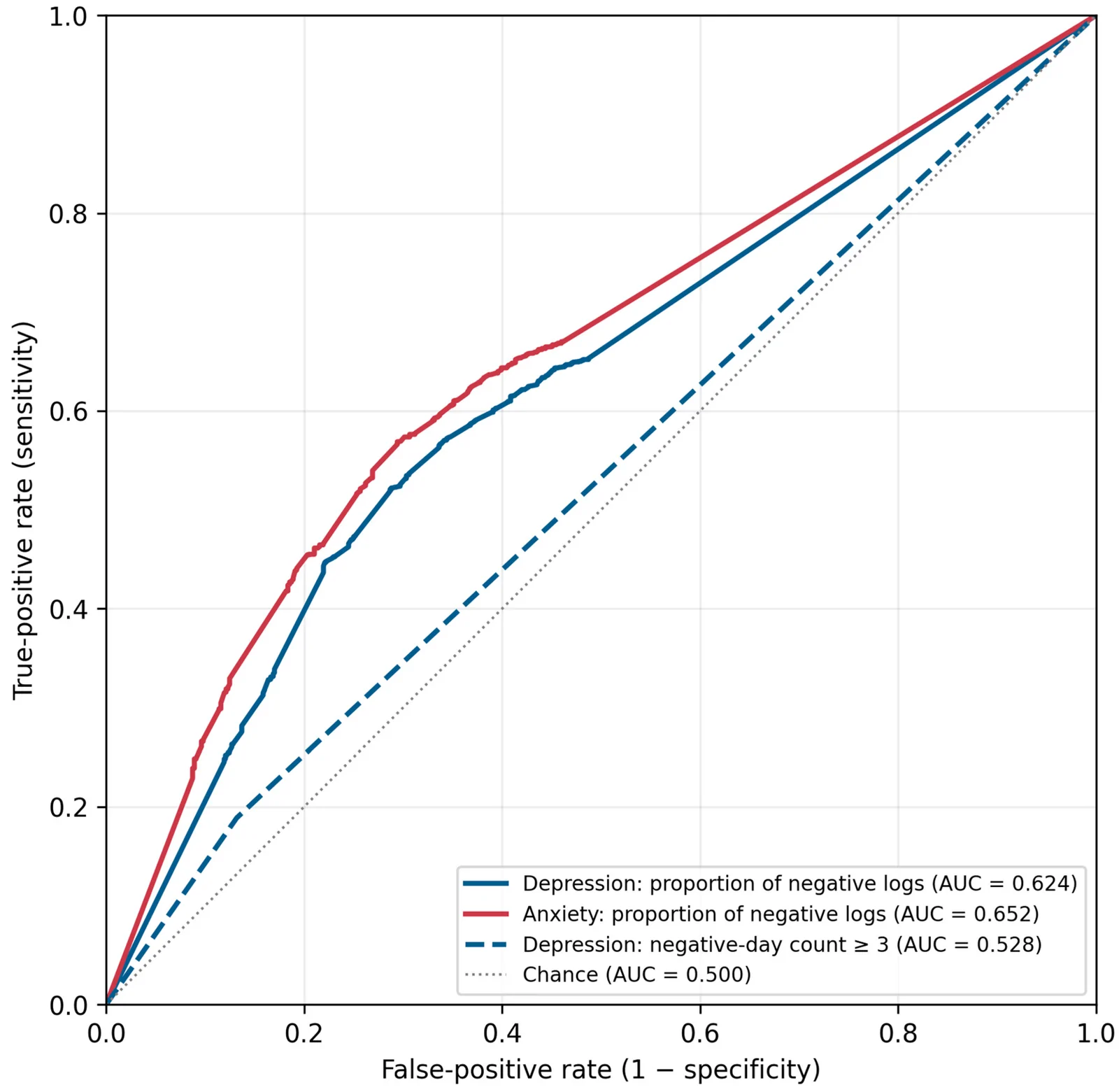
ROC curves for the primary candidate mood indices against binary reference-standard positivity at the raw ≥ 10 threshold on the validated Arabic PHQ-9 (depression) and Arabic GAD-7 (anxiety). AUCs with bootstrap 95% CIs: proportion of negative logs—depression 0.624 (0.604–0.642), anxiety 0.652 (0.629–0.676); negative-day count ≥3 (depression) 0.528 (0.514–0.542). Adults aged 18 years or older. None of the curves reach the conventional AUC ≥ 0.80 benchmark for a clinically useful screening test.

The best-performing index for both constructs was neg_mood_ratio. For depression, AUC = 0.624 (95% CI 0.604–0.642); for anxiety, AUC = 0.652 (95% CI 0.629–0.676). Youden-optimal operating characteristics with bootstrap 95% CIs were: depression sensitivity 0.522 (0.506–0.538), specificity 0.712 (0.679–0.741), PPV 0.888 (0.874–0.901), NPV 0.254 (0.235–0.272), LR+ 1.81 (1.62–2.03), LR− 0.67 (0.64–0.71); anxiety sensitivity 0.569 (0.547–0.589), specificity 0.706 (0.670–0.743), PPV 0.867 (0.848–0.886), NPV 0.327 (0.302–0.353), LR+ 1.93 (1.71–2.22), LR− 0.61 (0.57–0.66). PPVs of 0.87–0.89 and NPVs of 0.25–0.33 reflect the reference-standard prevalence ceiling rather than test properties per se. Spearman rank correlations with continuous score percentage were 0.232 for depression (95% CI 0.204–0.262) and 0.268 for anxiety (95% CI 0.231–0.305); both *p* < 0.001. The corresponding ROC curves are shown in Figure 2.

**Table 1:** Diagnostic-accuracy properties of candidate mood indices against binary reference-standard positivity (raw score ≥10), with bootstrap 95% CIs.

Construct	Index	AUC (95% CI)	Sens (95% CI)	Spec (95% CI)	PPV	NPV	LR+ (95% CI)
**Depression**	Proportion negative logs	0.624 (0.604–0.642)	0.522 (0.506–0.538)	0.712 (0.679–0.741)	0.888	0.254	1.81 (1.62–2.03)
**Depression**	Dominant-negative (binary)	0.609 (0.592–0.625)	0.441 (0.424–0.457)	0.777 (0.749–0.805)	0.896	0.241	1.97 (1.74–2.26)
**Depression**	Mood-label entropy	0.537 (0.516–0.556)	0.378 (0.361–0.393)	0.712 (0.683–0.740)	0.852	0.207	1.31 (1.18–1.47)
**Depression**	Negative-day count ≥ 3	0.528 (0.514–0.542)	0.189 (0.176–0.202)	0.868 (0.842–0.891)	0.862	0.196	1.43 (1.18–1.74)
**Anxiety**	Proportion negative logs	0.652 (0.629–0.676)	0.569 (0.547–0.589)	0.706 (0.670–0.743)	0.867	0.327	1.93 (1.71–2.22)
**Anxiety**	Dominant-negative (binary)	0.620 (0.600–0.640)	0.427 (0.406–0.450)	0.813 (0.778–0.844)	0.885	0.297	2.28 (1.90–2.77)
**Anxiety**	Mood-label entropy	0.535 (0.509–0.560)	0.475 (0.452–0.497)	0.624 (0.582–0.663)	0.809	0.261	1.26 (1.13–1.42)
**Anxiety**	Negative-day count ≥ 3	0.537 (0.520–0.553)	0.219 (0.200–0.239)	0.854 (0.824–0.881)	0.835	0.245	1.50 (1.23–1.87)

Adults aged 18 years or older. Bootstrap 95% CIs from 1000 resamples. Sens/Spec/PPV/NPV/LR+ at Youden-optimal threshold. None of the primary indices reached the conventional AUC ≥ 0.80 benchmark; LR+ values below or at the lower end of the Jaeschke et al. (1994) [20] 2–5 range indicate diagnostically limited post-test-probability shift.

None of the primary indices reached the 0.80 benchmark for a clinically useful screening test. Positive likelihood ratios of 1.81 and 1.93 fall below the 2–5 range [20] that produces even a small clinically meaningful shift in post-test probability.

### 3.3. Regression on Continuous Scores

For depression, the coefficient for the z-scored neg_mood_ratio was +5.32 score-percentage points per standard deviation (*p* < 0.001), adjusted for age (β = −4.78 per decade, *p* < 0.001), male sex (β = −2.00, *p* = 0.022), and iOS device (β = 0.35, *p* = 0.73); adjusted R^2^ = 0.082; logistic-model out-of-fold AUC = 0.669. For anxiety, the coefficient was +6.59 score-percentage points per standard deviation (*p* < 0.001); adjusted R^2^ = 0.096; logistic out-of-fold AUC = 0.665. Odds ratios for the z-scored neg_mood_ratio were 1.65 (95% CI 1.50–1.82; depression) and 1.91 (1.68–2.16; anxiety); E-values (square-root approximation for a common outcome) of 1.75–1.92 (95% CI bound) and 1.89–2.11 (point estimate) indicate that an unmeasured confounder would need moderate—not overwhelming—joint association strength with both exposure and outcome to fully explain the associations away, under the E-value framework’s stated assumptions.

### 3.4. Temporal Ordering of Mood Logs Relative to Reference-Standard Completion

Defining the scale-to-log lag as (mood-log date—reference-standard-scale-completion date), positive lag values denote mood logs recorded after the scale and negative values denote logs before. In the depression-intersection cohort, the log-level distribution of lag days across all mood logs from all users was heavily right-skewed: median 19 days (IQR 0 to 116 days), mean 62.6 days (SD 152.9), range −673 to +806; 80.9% of all mood logs were recorded on or after the scale-completion day. In the anxiety-intersection cohort, corresponding values were median 25 days (IQR 0 to 132), mean 71.5 days (SD 166.8), range −675 to +815; 79.4% of mood logs were on or after the scale-completion day.

Consequently, restricting the index to logs recorded before scale completion retained only a minority of users. Among users with at least one pre-completion log, the proportion of negative logs gave AUC = 0.586 (95% CI 0.554–0.618; n = 1231) for depression and 0.605 (0.569–0.641; n = 855) for anxiety; restricting to logs within 30 days before completion gave 0.584 (0.552–0.615; n = 1083) and 0.605 (0.569–0.643; n = 779), and within 7 days 0.576 (0.542–0.610; n = 938) and 0.594 (0.557–0.629; n = 683). These values are lower than the all-time estimates, but their confidence intervals exclude 0.50. When all cohort members were retained and users without pre-completion logs were assigned a value of zero, AUCs were 0.497–0.504 for depression and 0.507–0.513 for anxiety, with all 95% CIs including 0.50, because most users had no pre-completion log. This pattern is stated here as a descriptive sequencing observation; the Discussion presents three plausible mechanisms in parallel—post-completion self-monitoring, therapeutic reflection during ongoing care, and shared platform-engagement propensity—without privileging a causal-dependence interpretation.

### 3.5. Discriminant Validity

In the both-scales cohort (N = 1825), the between-construct correlation ρ(depression score, anxiety score) = 0.575, indicating substantial comorbidity, as expected. The depression-specific composite (Sad + Frustrated + Tired ratio) correlated with depression score at ρ = 0.265 and with anxiety score at ρ = 0.206; Steiger’s Z for the within-versus-between difference = 2.82, *p* = 0.005—supporting genuine, if modest, separability of the depression signal from the anxiety signal at the level of dependent correlations. The anxiety-specific single-label (Anxious ratio) correlated with anxiety score at ρ = 0.066 and with depression score at ρ = 0.060; Steiger’s Z = 0.32, *p* = 0.75—no separable anxiety-specific signal, attributable to the anxiety-label prevalence of only 2.24% of all logs on this platform (a platform-specific feature of Talbinah’s label taxonomy rather than a general property of Arabic-language mood-tracking).

### 3.6. Simplicity Paradox—The Robustness Sweep (Table 2)

Thirteen comparator index specifications plus four machine-learning models were tested. No specification reached AUC ≥ 0.70 for either construct within the pre-specified feature space and model family. Between-index AUC comparisons via bootstrap ΔAUC 95% CIs against the primary neg_mood_ratio specification are reported in Table 2. Illustratively, the neg_mood_ratio versus negative-day-count- ≥ 3 comparison for depression yielded ΔAUC = −0.096 (bootstrap 95% CI −0.115 to −0.077, *p* < 0.001; alternative minus primary)—the primary index is statistically significantly better than this alternative.

**Table 2:** Ranked robustness sweep—Top specifications for each construct, with bootstrap ΔAUC 95% CI against primary specification.

Construct	Specification	AUC (Bootstrap 95% CI)	ΔAUC vs. Primary (95% CI)	*p* (DeLong-Equivalent)
**Depression**	Proportion negative logs (primary)	0.624 (0.604–0.642)	— (ref)	—
**Depression**	XGBoost (out-of-fold)	0.629 (0.609–0.649)	+0.005 (−0.006 to +0.019)	0.34
**Depression**	Logistic + interactions (out-of-fold)	0.624 (0.605–0.644)	0.000 (−0.013 to +0.014)	0.98
**Depression**	Dominant-negative binary	0.609 (0.592–0.625)	−0.015 (−0.026 to −0.004)	<0.001
**Depression**	Depression-specific composite (Sad + Frust + Tired)	0.614 (0.595–0.632)	−0.010 (−0.019 to −0.001)	0.04
**Depression**	Weighted-negative composite	0.626 (0.607–0.647)	+0.002 (−0.002 to +0.007)	0.34
**Depression**	Mood-label entropy	0.537 (0.516–0.556)	−0.087 (−0.111 to −0.064)	<0.001
**Depression**	Negative-day count ≥ 3	0.528 (0.514–0.542)	−0.096 (−0.115 to −0.077)	<0.001
**Anxiety**	Proportion negative logs (primary)	0.652 (0.629–0.676)	— (ref)	—
**Anxiety**	XGBoost (out-of-fold)	0.648 (0.624–0.674)	−0.003 (−0.017 to +0.010)	0.64
**Anxiety**	Dominant-negative binary	0.620 (0.600–0.640)	−0.031 (−0.045 to −0.018)	<0.001
**Anxiety**	Weighted-negative composite	0.652 (0.628–0.675)	+0.001 (−0.005 to +0.005)	0.85
**Anxiety**	Anxiety-specific single label (Anxious)	0.544 (0.528–0.560)	−0.108 (−0.132 to −0.084)	<0.001
**Anxiety**	Anxiety-specific composite (Anxious + Frust)	0.585 (0.566–0.605)	−0.066 (−0.087 to −0.045)	<0.001
**Anxiety**	Mood-label entropy	0.535 (0.509–0.560)	−0.117 (−0.144 to −0.089)	<0.001
**Anxiety**	Negative-day count ≥ 3	0.537 (0.520–0.553)	−0.115 (−0.139 to −0.090)	<0.001

Bootstrap ΔAUC 95% CIs from 1000 paired resamples on same subjects (DeLong-equivalent). Cross-validated AUCs for machine-learning models from 5-fold StratifiedKFold with within-fold preprocessing and hyperparameter tuning; LightGBM, Random Forest, and (for anxiety) logistic regression with interactions results are reported in Section 3.6. No machine-learning model improved on the primary index (all ΔAUC 95% CIs include zero)—consistent with the simplicity paradox. No specification reached AUC ≥ 0.70 for either construct. Adults aged 18 years or older. (ref), reference (primary) specification.

No machine-learning model improved on the primary index. For depression, out-of-fold AUCs were 0.629 (XGBoost; ΔAUC +0.005, 95% CI −0.006 to +0.019, *p* = 0.34), 0.628 (LightGBM; +0.004, −0.009 to +0.018, *p* = 0.50), 0.624 (Random Forest; 0.000, −0.011 to +0.012, *p* = 0.85) and 0.624 (logistic regression with interactions; 0.000, −0.013 to +0.014, *p* = 0.98). For anxiety, they were 0.648 (XGBoost; −0.003, −0.017 to +0.010, *p* = 0.64), 0.641 (LightGBM; −0.011, −0.027 to +0.004, *p* = 0.22), 0.653 (Random Forest; +0.002, −0.011 to +0.015, *p* = 0.84) and 0.636 (logistic regression with interactions; −0.015, −0.032 to +0.002, *p* = 0.08). Training-fold AUCs exceeded out-of-fold AUCs by 0.04–0.11, indicating that the added model complexity fitted the training folds without improving out-of-fold discrimination. This is a bounded empirical observation about this feature space and model family, not an unbounded empirical ceiling claim about what any model could achieve on this data structure.

Simplicity-paradox finding. The weighted-negative composite specification, which changed overall AUC only marginally (+0.002 for depression), destroyed discriminant validity: re-running the Steiger Z test in the both-scales cohort with the weighted composite in place of the depression-specific composite yielded Z = 1.18, *p* = 0.24—the depression signal was no longer separable from the anxiety signal. Folding the Anxious label into the composite pulled it toward a general-distress dimension shared by both constructs. This is a specific and reportable methodological caution, observed here on the Talbinah platform’s small nine-label Arabic mood-label taxonomy; whether the same pattern replicates on Arabic-language single-label EMA data with different taxonomies, densities, and populations is a hypothesis for replication rather than an established general property of single-label EMA.

### 3.7. Sensitivity Analyses

Complete-case and missing-indicator regression coefficients for the primary index differed by less than 0.1 score-percentage points (depression 5.32 vs. 5.34; anxiety 6.59 vs. 6.65). First-attempt, last-attempt, and mean-of-all-attempts reference-standard specifications yielded Spearman correlations of 0.232–0.244 (depression) and 0.268–0.275 (anxiety). Threshold sensitivity (raw ≥10 versus platform severity ≥40%) moved AUC by less than 0.01 for either construct (0.624 vs. 0.624; 0.652 vs. 0.656). The pre-completion sensitivity analyses are reported in Section 3.4. Primary confirmatory findings survive Bonferroni correction (Spearman *p* < 10^−52^ depression, *p* < 10^−40^ anxiety, against a Bonferroni-corrected α of 0.05/30 = 0.0017); smaller index-level differences identified in the robustness sweep should be read as exploratory rather than confirmatory.

## 4. Discussion

### 4.1. Principal Findings

A note on retrospective association versus prospective screening. The primary diagnostic-accuracy analyses in this study characterise retrospective, cross-sectional convergence between mood-log profiles and reference-standard screening-positivity; they do not characterise the prospective screening performance of mood-log profiles for as-yet-unassessed depressive or anxious symptoms. This distinction is central to the interpretation of every effect estimate reported below and follows directly from the temporal ordering documented in Section 3.4: approximately 80% of mood logs in the intersection cohorts (80.9% in the depression subset; 79.4% in the anxiety subset) were entered on or after the reference-standard scale had already been completed, with mean scale-to-log lags of +62.6 days (depression) and +71.5 days (anxiety). The pooled all-time AUC estimates in Table 1 and Table 2 therefore answer a retrospective, cross-sectional question: How well does a user’s overall mood-log profile track their earlier or contemporaneous formal-scale screening-positivity? They do not answer the prospective question: How well would a user’s mood logs, observed in advance of formal assessment, forecast their future screening-positivity? The closest available approximation to the prospective question is the pre-completion sensitivity analysis (Section 3.4), which restricts the mood-index computation to logs entered strictly before the reference-standard scale. Among users who had such logs, discrimination was weaker than for the pooled all-time specification (AUC 0.576–0.586 for depression and 0.594–0.605 for anxiety, all 95% CIs above 0.50); when users without pre-completion logs were retained and scored zero, discrimination was at chance level (AUC 0.497–0.513). Even this pre-completion signal is small, is confined to the minority of users who log mood before formal assessment, and remains a retrospective estimate rather than a measure of prospective screening performance. This reinforces that the platform’s dominant use pattern is retrospective confirmation of, rather than prospective anticipation of, a formal screening-positive profile.

In a large linked-records cohort from a Saudi Arabic-language digital mental health platform, naturalistic single-label mood tracking showed a statistically reliable but modest-in-magnitude retrospective association with the validated Arabic PHQ-9 (depression) and Arabic GAD-7 (anxiety) reference standards. The relationship did not reach the conventional 0.80 AUC benchmark commonly cited in the diagnostic-accuracy literature for a clinically useful stand-alone screening test, and the positive likelihood ratios of the best-performing index fell below the 2–5 range that Jaeschke et al. (1994) [20] associate with even small clinically meaningful shifts in post-test probability. Single-label mood tracking on this platform should therefore not substitute for the validated Arabic PHQ-9 or Arabic GAD-7. Beyond this headline negative screening result, three structural findings emerged. First, the majority of mood logs on this platform were entered on or after the reference-standard scale had been completed rather than before it, an ordering that is a property of user behaviour on the platform rather than an artefact of the analytic design. Second, a depression-specific composite (Sad + Frustrated + Tired ratio) discriminated depression from anxiety with a Steiger’s Z of 2.82 (*p* = 0.005) in the both-scales subset, while the analogous anxiety-specific single label (Anxious) did not (Z = 0.32, *p* = 0.75), reflecting the very low 2.24% platform-level prevalence of the Anxious label. Third, adding complexity—through more labels, weighted composites, or machine-learning models—either failed to raise the cross-validated AUC or destroyed discriminant validity.

### 4.2. Comparison with Prior Work

Prior single-label EMA and mood-tracking work has largely characterised engagement and satisfaction outcomes [7,8] rather than evaluated screening-positive detection accuracy against a validated reference standard. Studies of that type are especially rare in non-English-language contexts. To our knowledge, this is the first study to evaluate screening-positive detection accuracy of single-label naturalistic mood tracking against the validated Arabic PHQ-9 and Arabic GAD-7 in a Saudi Arabic-language digital mental health cohort. Related work on individual-level digital behavioural markers of mental illness (e.g., Chen et al. 2022 [23]) has proposed a growing set of app- and sensor-derived signals as candidate biomarkers, but has not tested single-label naturalistic EMA-against-validated-reference-standard screening-positive detection accuracy on an Arabic-language platform. The present findings sit within—and refine—this literature: naturalistic single-label mood logs on this specific Saudi platform carry a modest but non-zero retrospective convergent-validity signal on continuous scores, but do not reach the operating characteristics required to substitute for a validated instrument.

### 4.3. Clinical and Platform-Design Implications

On this specific Saudi Arabic-language platform, single-label mood tracking should not be operated as a stand-alone screening indicator for depression or anxiety, and its clinical value is best positioned as a longitudinal engagement and self-monitoring indicator rather than a screening substitute for the validated Arabic PHQ-9 and Arabic GAD-7 which the platform already administers. The temporal-sequencing observation (mood logs recorded predominantly on or after the scale-completion day) is descriptively consistent with mood tracking functioning as a post-completion self-monitoring or therapeutic-reflection feature, but it does not, on its own, establish causal dependence—three mechanisms are plausible (post-completion self-monitoring; therapeutic reflection during ongoing care; and shared platform-engagement propensity across features), and the present analysis cannot distinguish among them. Broader inferences (applicability to “platform designers”, “Arab-language platforms”, or “mental health technology investors”) should be reframed as hypotheses requiring independent verification on other Arabic-language digital mental health platforms with different label taxonomies, populations, and workflows.

### 4.4. Strengths and Limitations

Strengths. To the author’s knowledge, this is the first study to evaluate the accuracy of naturalistic single-label Arabic mood tracking for detecting screening-positive scores on the validated Arabic PHQ-9 and Arabic GAD-7 in a large linked-records commercial-platform cohort. The pre-specified robustness sweep across alternative index specifications and machine-learning models, the bootstrap 95% confidence intervals on every operating characteristic and every ΔAUC comparison, and the transparent report of the negative screening result together support the paper’s methodological contribution.

Limitations. Several important limitations should be considered when interpreting the findings. First, the study is a retrospective secondary analysis of a single commercial platform in Saudi Arabia; the specific magnitudes require replication elsewhere on other Arabic-language platforms with different label taxonomies and populations. Second, the very high reference-standard positivity (81.4% depression, 77.1% anxiety) reflects a spectrum effect: platform users who self-initiate a formal-scale completion are enriched for symptomatic individuals relative to the general population. Because PPV and NPV are strongly prevalence-dependent, they will not generalise directly to differently selected populations; sensitivity, specificity, and likelihood ratios (all reported in Table 1 with bootstrap 95% CIs) are more portable indices for readers wishing to compare across settings. Third, item-level PHQ-9/GAD-7 responses were not accessible, so item-level analyses of screening-positive detection accuracy (Set D) were not possible. Fourth, the Arabic GAD-7 version deployed on the platform has been psychometrically validated in a Lebanese Arabic-speaking primary-care sample (Sawaya et al. 2016 [13]) but not in a Saudi-specific validation study; the cross-national portability of the Arabic GAD-7 psychometrics from Lebanon to Saudi Arabia is therefore an inference, not an established property, and results should be interpreted with this in mind. Fifth, the platform’s small label taxonomy—and specifically the very low 2.24% prevalence of the “Anxious” label—limits inferences about anxiety-specific single-label EMA to platforms with similar taxonomies. Sixth, the covariate adjustment set (age, sex, device type) is limited; it does not control for symptom severity trajectory, prior treatment, psychosocial stressors, or onboarding-funnel effects, and the E-values (1.75–2.11) quantify but do not eliminate this residual-confounding concern. Seventh, a positive screen at the raw ≥10 threshold is not a clinical diagnosis and requires clinician confirmation to be actionable. Eighth, the platform’s Terms of Service consent framework is a lower-friction consent model than opt-in specific consent; a stricter opt-in consent model would be preferable for identifiable data, and this is a genuine ethical limitation of secondary-research use of platform-held records. Ninth, the analyses were pre-specified per an internal written analysis protocol drafted before the analyses were executed; there was no public pre-registration on a timestamped registry, and readers should therefore read the smaller index-level differences in the robustness sweep as exploratory rather than confirmatory; notably, none of the machine-learning models improved on the primary index (all ΔAUC 95% CIs included zero). Tenth, users aged 10–17 years were excluded, so the findings apply to adult users only.

## 5. Conclusions

Single-label naturalistic Arabic mood tracking on a large Saudi Arabic-language digital mental health platform shows a modest but statistically reliable retrospective association with screening-positive status on the validated Arabic PHQ-9 and Arabic GAD-7 that nevertheless falls short of the operating characteristics required for a stand-alone screening test. It should not substitute for these validated instruments. Beyond this headline negative screening result, three findings warrant methodological attention. First, the temporal distribution of mood logs on this platform is heavily skewed toward the post-completion window, and this pattern shapes the interpretation of every convergent-validity estimate that pools all-time mood logs: among users who had logs recorded before formal assessment, those logs carried weaker discrimination (AUC 0.58–0.61), so these estimates describe retrospective association rather than prospective screening performance. Second, the depression signal is genuinely separable from the anxiety signal at a simple composite level but only when composite complexity is minimised. Third, adding index or model complexity either fails to improve or actively harms discriminant validity—a simplicity paradox that should temper enthusiasm for machine-learning approaches to naturalistic mood-log EMA in the single-label Arabic setting studied here.

Scope of the present evidence. The empirical base for these conclusions is a single Arabic-language commercial digital mental health platform operating in Saudi Arabia between 2023 and 2026, with a specific single-label EMA architecture (nine user-initiated emoji-labelled categories displayed on each app open) and a specific adult (18 years or older), predominantly young, female and iOS-majority user base. Extrapolation of these screening-accuracy estimates to other Arabic-language digital mental health platforms that use different mood-label taxonomies or prompted rather than user-initiated EMA architectures, to non-Arabic-language platforms serving other Muslim-majority or non-Muslim-majority populations, or to clinical populations accessing traditional in-person mental health services is not warranted on the present evidence and should be treated as an empirical research question rather than an assumption. Similarly, the temporal-ordering, discriminant-validity, and simplicity-paradox findings reported here are methodological observations of the specific single-label EMA implementation studied; whether they generalise across implementations is itself a testable hypothesis for future replication studies.

## Data Availability

The de-identified dataset is held by the Talbinah platform under a data-governance agreement and is not publicly available. Aggregated results, tables, and analytical outputs are provided in full within this manuscript. The analytic code and pipeline (Python scripts implementing the four primary indices, the robustness sweep of alternative specifications, the four machine-learning models, DeLong-bootstrap AUC-difference testing, and Steiger’s Z discriminant-validity test) are available upon reasonable request from the corresponding author.

## Supplementary Materials

The following supporting information can be downloaded at: https://www.mdpi.com/article/10.3390/diagnostics16213560/s1, Table S1: Characteristics of the adult analytic cohort and the three intersection cohorts.
