# Supplementary material for: When Users Log Mood After Assessment: Temporal-Lag, Discriminant Validity, and the Simplicity Paradox of Single-Label Ecological Momentary Mood Tracking in a Saudi Arabic-Language Digital Mental Health Cohort—A Diagnostic-Accuracy Study
Source: Diagnostics (Basel). 2026 Oct 29;16(21):3560. doi: 10.3390/diagnostics16213560 (PMC13762194; doi:10.3390/diagnostics16213560)
Supplement: Supplementary file 1 [file diagnostics-16-03560-s001.zip › diagnostics-4563113-supplementary.pdf]

Supplementary Table S1. Characteristics of the adult analytic cohort and the three intersection cohorts.

Cohort	n	Age, years, mean (SD)	Female, %	Device, %: iOS / Android / other / missing	Mood logs per user, median (IQR)
Adult analytic cohort	25,555	28.8 (8.9)	70.8	80.1 / 15.6 / 0.6 / 3.7	2 (1–3)
Depression cohort	4,236	27.6 (8.0)	77.7	83.2 / 13.2 / 0.7 / 2.9	3 (1–6)
Anxiety cohort	2,449	27.2 (7.8)	78.8	81.7 / 12.6 / 0.9 / 4.7	3 (1–9)
Both-scales cohort	1,825	27.1 (7.7)	80.5	81.9 / 13.4 / 1.1 / 3.6	3 (2–9)

Adults aged 18–100 years. The adult analytic cohort comprises all cleaned mood-logging users aged 18 years or older; intersection cohorts additionally completed the Arabic PHQ-9 (depression), the Arabic GAD-7 (anxiety), or both.
